# *In Vivo* and *in Vitro* Study on Drug-Drug Interaction of Lovastatin and Berberine from Pharmacokinetic and HepG2 Cell Metabolism Studies

**DOI:** 10.3390/molecules21040464

**Published:** 2016-04-08

**Authors:** Hanming Cui, Jialong Wang, Qiuyan Zhang, Mengmeng Dang, Hui Liu, Yu Dong, Lu Zhang, Fang Yang, Jianhua Wu, Xiaolin Tong

**Affiliations:** 1 Guang’anmen Hospital, China Academy of Chinese Medical Sciences, Beijing 100053, China; footprint666@163.com (J.W.); piaoliumu828@163.com (Q.Z.); dongyu250541@sina.com (Y.D.); 2 College of Pharmacy, Shannxi University of Traditional Chinese Medicine, Xianyang 712046, China; danseshijue1206@163.com (M.D.); 13279563002@163.com (H.L.); wu0700@163.com (J.W.); 3 College of Pharmacy, Tianjin University of Traditional Chinese Medicine, Tianjin 300193, China; 13920026618@163.com; 4 Department of Agriculture and Life Sciences, Ankang University, Ankang 725000, China; akxyyf@163.com

**Keywords:** *in vivo* and *in vitro*, berberine, lovastatin, pharmacokinetics, HepG2 cell

## Abstract

*Background*: We assumed that the pharmacokinetics of lovastatin could be changed by the induction effect of berberine. *Methods*: An UPLC-MS/MS method was developed and validated for the pharmacokinetics tudy of lovastatin to investigate the *in vivo* drug-drug interactions between lovastatin and berberine. SD male rats were random divided into lovastatin group and berberine induced prior to lovastatin group for the *in vivo* pharmacokinetic studies. Meanwhile HepG2 cells were induced by berberine for three days to study the metabolism of lovastatin. *Results*: The AUC (*p* < 0.01) and C_max_ (*p* < 0.01) could be significantly decreased in the berberine-induced group *in vivo*, and the metabolic activity of HepG2 cell ccould be increased by berberine induction *in vitro*. The metabolism parameters of lovastatin such as CL, V_max_ and K_m_ were increased after the induction of berberine. From the pharmacokinetic study of lovastatin induced with berberine, we obtained pharmacokinetic parameters which are compliance with the metabolic parameters of lovastatin in HepG2 cells with berberine induction *in vitro*. *Conclusions*: From the *in vivo* pharmacokinetics study and the HepG2 cell metabolism study *in vitro*, berberine could be an inducer for the metabolism of lovastatin according to our previous research on berberine induction effects on HepG2 cells, which may be relevant to the fact that berberine possesses induction effects through the CYP 450 3A4 enzyme.

## 1. Introduction

**Figure 1:**
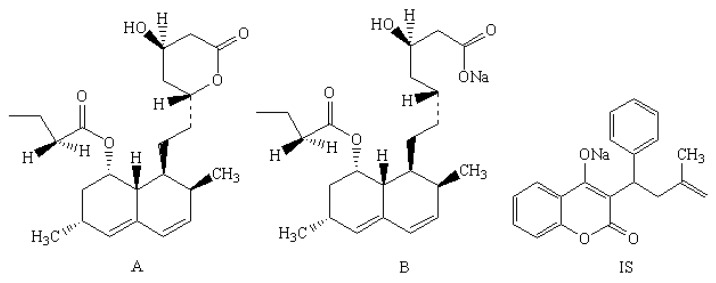
A, B and IS are the chemical structures of lovastatin, lovastatin acid and warfarin sodium.

High cholesterol and diabetes-related diseases are urgent healthy problems with an incidence of more than 10% among the Chinese population [1]. Clinical therapeutics in this area involves prescription of the herbal medicines *Monascus* and *Coptis* *Chinensis* Franch, which contain the main bioactive components of berberine and lovastatin. Lovastatin is a cholesterol-lowering drug isolated from *Monascus* and astrain of *Aspergillusterreus* [2,3,4,5], that competitively inhibits the biosynthesis of mevalonic acid by HMG-Co Areductase. This key enzyme catalyzes the conversion of HMG-CoA to mevalonate. An early and rate-limitings tep in the biosynthesis of cholesterol. After oral administration, an inactive lactoneis hydrolyzed by carboxyesterases to the corresponding β-hydroxy acid (Figure 1), where the hydrolyzate is considered to be the main metabolite and active pharmaceutical ingredient of lovastatin [6,7,8,9].

The literature has reported drug-drug interactions between lovastatin and drugs or related products with effect on drugs such as colesevelam, gemfibrozil, itraconazole or dietary supplements determined via pharmacokinetic studies [10,11,12,13,14,15]. Berberine is an alkaloid available from the medicinal plant *Coptis* *chinensis* that is widely used clinically in the treatment of gastrointestinal infections, diabetes, hypertension, and hypercholesterolemia. There are numeros drug-drug interaction studies related to berberine with Chinese medicine or drugs such as Jiao-Tai-Wan, verapamil, flavonoids of radix Scutellariae, digoxin, simvastatin, ciprofloxacin, fluconazole, doxorubicin, pravastatin, rhein, cyclosporine A, ketoconazole, losartan, probenecid, cloxacillin, midazolam, quinidine and atorvastatin [16,17,18,19,20,21,22,23,24,25,26,27,28,29]. Combination of simvastatin with berberine improves the lipid-lowering efficacy [20]. Our previously study proved that berberine is an inducer in the CYP 450 3A4 enzyme metabolism by increasing the RNA transcription [30,31,32], and berberine potentially has a drug-drug interaction which affects the pharmacokinetics of lovastatin in rats. Therefore, the study of the pharmacokinetics of lovastatin acid after a single dose of lovastatin, or berberine-induced rats prior to lovastatin administration has great significance in the therapeutical control of cholesterol-lowering drugs in the clinic to reduce the adverse effects.

As the literature has reported, lovastatin acid sodium was selected as the analyte due to its greater sensitivity than lovastatin in MS/MS detection [33,34]. In the present study, we developed an UPLC-MS/MS method for the determination of lovastatin acid sodium in rat plasma with LOQ at 0.25 ng·mL^−1^, which is more sensitive than the previously reported LOQ of 1 nM for lovastatin acid. In the application of the validated bio-analysis method to the study of the lovastatin pharmacokinetics induced by berberine, SD male rats were randomly divided into two groups (lovastatin group and berberine-induced lovastatin group). The plasma samples were collected on a schedule and prepared for analysis. Evaluation of the drug-drug interactions between lovastatin and berberine was performed by comparing the significant difference of the main pharmacokinetics parameters between the lovastatin group and berberine-induced lovastatin group. Meanwhile, a comparative evaluation of the metabolic activity of lovastatin was performed in the HepG2 cell line between the lovastatin group and the berberine-induced lovastatin group as well.

The results showed that the validated method could be successfully applied to study the drug-drug interactions between lovastatin and berberine. After induction of berberine, pharmacokinetic parameters such as the AUC (*p* < 0.01) and C_max_ (*p* < 0.01) were significantly decreased *in vivo*. In the *in vitro* HepG2 cells experiments, berberine could increase the metabolic activity by increasing the metabolic parameters of CL, which was increased from 1.06 to 1.48 mL/10^5^(cell)·h^−1^ with the induction of berberine, the K_m_ was increased three times in berberine induced group as well, which was compliance with the obtained pharmacokinetics parameter from AUC_0–12_ of lovastatin decreased 2.5 times (approximately three times).

## 2. Results

### 2.1. UPLC-MS/MS Method Development

The UPLC-MS/MS conditions were as follows: the mobile phases were 0.1% formic acid in water (A) and 0.1% formic acid in acetonitrile (B) with the following program profile: 0–1.2min, A (90%~85%) *vs.* B (10%~15%), 1.3–1.8min, A (50%~10%) *vs.* B (50%~90%), 2.1–2.5 min, A (10%) *vs.* B (90%), 2.5–2.9min, A (10%~90%) *vs.* B (90%~10%), 3.0–4.0 min, A (90%) *vs.* B (10%). The mobile phase was pumped at a flow rate of 0.3 mL·min^−1^, and the column temperature was 25 °C. The ESI source temperature was optimized at 500 °C, the nitrogen gas was 800 L/h. The multiple reaction monitoring model (MRM) was used to select the precursor ion, production, cone and collision voltage by Intellistart optimization for lovastatin acid and warfarin, with the results shown in Table 1.

### 2.2. Plasma Sample Procedure

Extraction of lovastatin acid from rat plasma was optimized by means of liquid-liquid extraction and protein precipitation. The result showed that lovastatin could betransformed into lovastatin acid with a recovery more than 89% at concentration levels of 1, 10 and 100 ng·mL^−1^, there was no obvious interference for lovastain acid extraction by the protein extraction method (recovery% ≥ 89.2%). For method validation, 100 μL of blank rat plasma was added into a 1.5 mL centrifuge tube, spiked with 10 μL working solution of IS and lovastatin acid respectively, then 10 μL of triethylamine was added and vortexed for 5 min. Then the mixture was extracted with 270 μL acetonitrile, vortexed eventually for protein precipitation for 5 min prior to centrifugation at 14,000× *g* for 15 min. Finally, 200 μL of the supernatant was placed into a 96 samples well plate for analysis. For true drug plasma procedure, 100 μL of plasma sample was spiked with 10 μL working solution of IS and 10 μL triethylamine prior to vortexing for 5 min, and then extracted with 280 μL acetonitrile for protein precipitation, vortexed for 5 min and centrifuged at 14,000× *g* for 15 min. Finally, 200 μL of the supernatant was transferred into a 96-well polypropylene plate for analysis.

### 2.3. Method Validation

**Figure 2:**
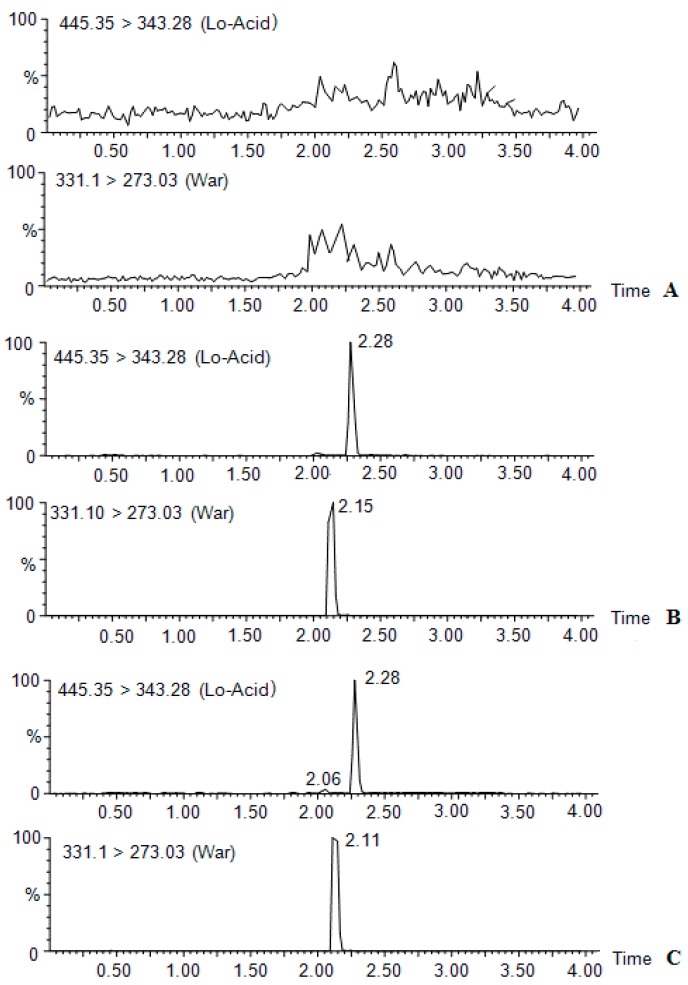
The chromatography for specification of lovastatin acid in rat plasma. (**A**–**C**) are the representation of the rat blank plasma, the reference spiked into blank plasma (1.0 ng·mL^−1^) and the true plasma samples at 1 h.

Method validation was strictly conducted according to the FDA guidance for method validation for bio-analysis [35]. For specification, the result showed that there was no interference with the analysis of lovastatin acid owing to the fact the blank showed no response at the sample retention time and quantification of MRM ions, while lovastatin acid could be determined after rat administration of lovastatin for 1 h (Figure 2). For LLOQ, six replicates were analyzed with the S/N ratio of 10 with the precision of RSD% less than 20% at concentration of 0.25 ng·mL^−1^. For linearity, lovastatin acid was linear at the concentration ranged from 0.25~100 ng·mL^−1^ with the correlation coefficient prior to 0.99 weighed by 1/x, the linear equations were y= (1.125 ± 0.12)x + (0.52 ± 0.15), where y is the relative response area of lovastatin acid *versus* the relative response area of IS, x is the concentration of lovastatin acid. The quality control was calculated according to the linear equation by accuracy in the range of 88.6%~105.4% during all the analysis. The inter-day and inner-day precision and accuracy were determined and the results showed that the precisions were in the range of 89.2%~105% with a relative standard deviation (RSD%) lessthan 10% at concentration levels of 1, 10 and 100 ng·mL^−1^. The RSD% for recovery, matrix effect and the stability were less than 10% at concentration levels of 1, 10 and 100 ng·mL^−1^ as well. All the method validation data, which is compliance with method validation for bio-analysis and approval for the pharmacokinetic study, is shown in Table 2.

### 2.4. Pharmacokinetic Study

**Figure 3:**
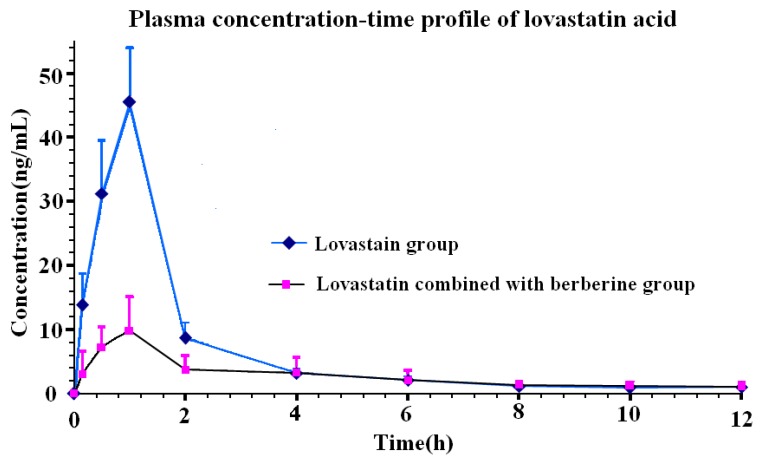
The drug plasma concentration (ng·mL^−1^)-time (hour) profile of lovastatin acid obtained from berberine induced lovastatin and lovastatin, respectively.

In the pharmacokinetic study, the drug plasma concentration-time profile for a rat’s single dose of lovastatin and berberine induced 3 days prior to dose of lovastatin is shown in Figure 3 (*n* = 6). The obtained pharmacokinetic compartment model was calculated according to minimum the value of Akaike’s Information Criterion (AIC), which pertains to the two-compartment model for the pharmacokinetic parameters calculation. The obtained first-order absorption constant (t_1/2α_) was 3.75 h for the berberine-induced group and 2.20 h for the lovastatin group, and the first-order elimination constant (t_1/2β_) was 16.60 h for the berberine-induced group and 2.93 h for the lovastatin group. The mean AUC_0–24_ for lovastatin acid was 91.97 ng/mL·h, the C_max_ was 47.40 ng·mL^−1^ and the *t*_max_ was 1 h. After berberine induction for 3 days, the means of AUC, C_max_ and *t*_max_ for lovastatin acid were 37.28 ng/mL·h, 11.13 ng·mL^−1^ and 0.92 h, respectively. When an independent sample T test was used for intergroup comparison to obtain the significant difference pharmacokinetic parameters between two groups, it was found that berberine could decrease the pharmacokinetic parameters such as C_max_, AUC_0–24_ and AUC_0-∞_ significantly (Table 3, *p* < 0.01), while the *t*_max_ was not delayed by the induction, and the mean retention time (MRT) and t_1/2β_ were prolonged.

### 2.5. Evaluation of Lovastatin Metabolism Induced by Berberine

**Figure 4:**
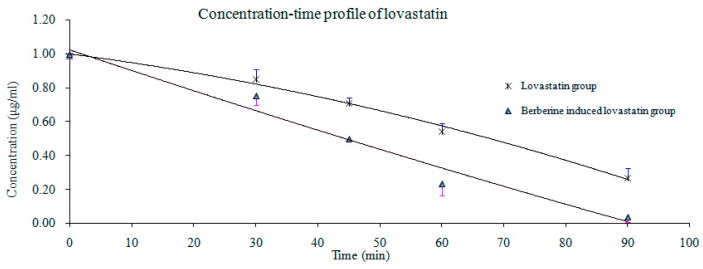
HepG2 cell metabolism of lovastatin plotted by drug concentration-time profile.

According to the UPLC-MS/MS analyzed data of lovastatin, the linearity and the QC meet the sample analysis requiremente for precision at the range of 90.5%~112.7%. The drug concentration time profile is shown in Figure 4, where the lovastatin metabolic rate was plotted with V (metabolicrate) *vs.* S (lovastatin concentration) according to the Michaelis-Menten equation V = V_max_ × S / (K_m_ + S), where V_max_ and K_m_ were calculated using the “Solver Options” of the Microsoft Excel software 2007, and the data was obtained from the lovastatin group and the berberine-induced group. The results showed that V_max_ and K_m_ for lovastatin were 6.60 ng/10^6^ (cell)·h^−1^ and 0.62 ng·mL^−1^ for a single dose in the lovastatin group, and the V_max_ and K_m_ were 19.94 ng/10^6^ (cell)·h^−1^ and 1.35 ng·mL^−1^ in the berberine-induced group. The intrinsic clearance of HepG2 was the ratio between V_max_ and K_m_ for lovastatin (CL), which were 1.06 and 1.48 mL/10^5^ (cell)·h^−1^ in the lovastatin group and berberine-induced group, respectively.

## 3. Discussion

Lovastatin is effective but has adverse effects on the cardiovascular system and risk factors for myopathy [5]. Consequently, the bio-availability study of lovastatin byAUC and C_max_ has great significance to reduce its adverse effects in the clinic when combined with berberine. This experimental study developed a highly sensitive and accurate UPLC-MS/MS method with a simple sample handling procedure for the pharmacokinetic study of lovastatin acid. Lovastatin acid is a hydrolysis product and active compound of lovastatin *in vivo*. Meanwhile, lovastatin acid is more sensitive than lovastatin for analysis on MS/MS, which is more accessible for the pharmacokinetic study of lovastatin. After induction of berberine for three days prior to administration of a single oral dose of lovastatin, the compartment model was evaluated as the two-compartment model. The pharmacokinetic parameter t_1/2β_ was significantly increased with the berberine induction, while the AUC and C_max_ were significantly decreased compared to the single oral dose of lovastatin. Those result revealed a potential drug-drug interaction between berberine and lovastatin.

In the HepG2 cells metabolism experiment, the results showed that the V_max_ of lovastatin was increased from 6.60 to 19.94 ng/10^6^ (cell)·h^−1^ after the induction with berberine, and the K_m_ was increased from 0.62 to 1.35 ng·mL^−1^ with the berberine induction as well. The intrinsic clearance CL was increased from 1.06 to 1.48 mL/10^5^ (cell)·h^−1^ with the induction of berberine. The HepG2 cell metabolism activity of lovastatin was increased significantly by the berberine induction, the K_m_ was increased three times in the berberine-induced group, which was compliance with the fact the AUC_0–12_ for lovastatin decreased 2.5 times (approximately three times), while the C_max_ of lovastatin decreased 2.5 times after induction with berberine. The data suggests that berberine may slow the adverse effects caused by continuous dosing of lovastatin.

Lovastatin is an substrate of the CYP 450 3A4 enzyme and P-glycoprotein [32,36], while berberine is an inducer of CYP 450 3A4 enzyme activity via an increase in its mRNA expression [30], but there is no mention in the literature of a relevant induction effect of berberine on *P*-glycoprotein. The metabolism parameter K_m_ increased rate, was less than the pharmacokinetic parameter C_max_ decreased rate, a result that could be explained by the absorption and metabolism of lovastatin with the involvement of P-glycoprotein and other CYP 450 enzymes. Our previous study found that berberine could increase CYP 450 3A4 enzymem RNA expression more than 2-fold *in vitro*, which was in agreement with the fact the AUC of lovastatin acid decreased more than 2-fold *in vivo*. According to the FDA guidance on drug development and drug interactions, berberine could decrease the AUC_0–t_ of lovastatin by 59.5%, which corresponds to a moderate inducer by decreasing the AUC in the range of 50%–80% by inducing the CYP 450 3A4 enzyme [37]. Accordingly, there is a literature report on a drug-drug pharmacokinetic interaction study of simvastatin combined with berberine after oral administration in rats [38], in which it was found that berberine could decrease the AUC_0-t_ of simvastatin acid by 60.9% (from 7258.3 ± 3995.6 μg/L·h decreased to 4421.7 ± 1384.3 μg/L·h). The pharmacokinetic study combined with the pharmacology research of simvastatin with berberine improvement of the lipid-lowering efficacy, suggest this may be a promising drug-drug combination to overcome its adverse effects and to improve its lipid-lowering efficacy inclinical use.

## 4. Experimental Section

### 4.1. Reagent and Chemicals

Acetonitrile and methanol of HPLC grade was purchased from Fisher Scientific (Fair Lawn, NJ, USA). Formic acid and ammonium acetate was purchased from TEDIA Inc. (Fairfield, OH, USA). Berberine and trimethylamine were obtained from Sigma Aldrich (Shanghai, China). Ultra purified water was prepared in our lab using a Milli-Q system (Millipore, Bedford, MA, USA).

Caco2 cell line was obtained from Cell Culture Center of Chinese Academy of Medical Sciences (Beijing, China). DMEM low glucose medium and fetal bovine serum were obtained from Gibco (GrandIsland, NewYork, NY, USA).

### 4.2. Instrumental

The UPLC-MS/MS system was consisted of a Waters Xevo Triple Quadrupole mass spectrometer equipped with an electrospray ionization source (ESI) (Waters, Milford, MA, USA), a quaternary pump, an UPLC Acquity BEH C_18_ column (2.1 × 50 mm, 1.7 μm) and the software Masslynx V 4.1 ChemStation.

### 4.3. Preparation of Reference Solution and Working Solutions

Lovastatin and warfarin sodium was purchased from the National Institutes for Food and Drug Control (Beijing, China). Lovastatin acid sodium was purchased from Merck Millipore (Calbiochem, Darmstadt, Germany). The working solutions for lovastatin acid were prepared in the range of 5~10,000 ng·mL^−1^ in a series concentration level. Warfarin sodium was selected as the internal standard (IS) and prepared at a concentration of 1000 ng·mL^−1^. All the standard solutions were stored at 4 °C in a refrigerator before use. The chemical structures of lovastatin, lovastatin acid sodium and warfarin sodium were shown in Figure 1.

### 4.4. Plasma Sample Procedure

Lovastatin transformation into lovastatinacid by basic hydrolysis was performed in the plasma sample procedure. The extraction of lovastatin acid from rat plasma procedures were optimized by means of liquid-liquid extraction by ethyl acetate added with formic acid or triethylamine and protein precipitation by methanol or acetonitrile added with formic acid or triethylamine.

### 4.5. UPLC-MS/MS Method Optimizing and Validation

The UPLC condition was optimized on an Acquity BEH C_18_ column for analysis. Selection of the mobile phase investigated the composition, flow rate, and addition of formic acid and ammonium acetate in various concentrations to obtain high sensitivity and ionization. The column temperature was 25 °C. The multiple reaction monitoring (MRM) model for parent to daughter ion (*m*/*z*) was used to optimize the energy of collision and cone by the procedure of Intellistart in combination with the mobile phase. The ESI source parameters were optimized with drying gas temperature and the gas flow rate as well.

The method validation includeds pecificity, linearity, lower limit of quantification (LLOQ), precession and accuracy. The specificity was to verify the interference from blank sample for the determination of IS and lovastatin acid with each retention time and MRM ions, drug-free rats’ plasma sample was collected and processed under sample procedure, and then analyzed according to the developed UPLC-MS/MS method. Linearity of the calibration curve prepared for a lovastatin acid concentration range of 0.25 to 100 ng·mL^−1^ in eight levels, was studied, using warfarin as internal standard. The LLOQ was based on the signal-to-noise (S/N) ratio of more than 10:1 and coefficient of variation less than 20% with multiple analysis (*n* = 6).

Quality control (QC) samples were prepared and analyzed at final concentrations of 0.5, 10 and 100 ng·mL^−1^ along with procedure samples at intervals of each run, which accuracy in the range of 80%~120% was considered to be a qualified analysis batch.

Precision and accuracy studies were conducted by spiking lovastatin acid and IS into matrices (drug-free blank plasma). Both the inner-assay and inter-assay (on three sequential day) reproducibility was studied to evaluate the precision and accuracy, using working solutions prepared at concentration levels of 1, 10 and 100 ng·mL^−1^.

Recovery and matrix effects were examined according to the sample procedure at concentration levels of 1, 10 and 100 ng·mL^−1^. Recovery was studied by preparing samples from prior-spiking and post-spiking of the standard into drug-free plasma to prepare samples, and the calculation was conducted by comparing the prior-spiking analysis to the post-spiking samples as a percentage (%). The matrix effect was determined by preparing samples from post-spiking of the standard into drug-free plasma to prepare samples, and then a calculation was conducted by the analysis of the post-spiking samples compared to the reference standard by percentage (%). For the transformation of lovastatinin to lovastatin acid, the production rate (percentage) was investigated as well as the recovery of lovastatin acid.

The stability was used to verify the stability of lovastatin acid in plasma. Drug-free samples were spiked with lovastatin acid, and then kept for 10 days, or a month in a frozen state for three melt-thaw cycles inrefriger at or under −40 °C prior to the sample procedures. The stability of the post procedure was investigated in the samples using the auto-sampler at room temperature.

### 4.6. Design of the Study of Lovastatin Pharmacokinetics Induced by Berberine

Commercial lovastatin capsules (20 mg, Lotnumber: 12101812) were obtained from Yangtze River Pharmaceutical Group Co., Ltd. (Taizhou, China). SD male rats (240 ± 20 g) were purchased from Beijing HFK Bioscience Co.Ltd. (license No: SCXK2009-0007, Beijing, China). The animals were maintained at ambient temperature and humidity on a 12 h light / 12 h dark cycle for a week before use, they were housed with free access to food and water except for fasting for 12 h. Animal household welfare and experimental procedures were strictly conducted according to the *Guidance* *For* *The* *Care* *and* *Use* *of* *Laboratory* *Animals* (Ministry of Science and Technology of the People’s Republic China, 2006) and the related ethical regulations of our hospital (Medical Ethics Committee of Guang’anmen Hospital). The ethics committee approved this work (permit number: 2013AEC001), and efforts were made to ameliorate animal suffering. The rats were randomly divided into two groups, one group was treated with the oral administration of lovastatin at the dose of 2 mg/kg calculated by body weight (*n* = 6), and another group was received berberine (10 mg/kg calculated by body weight) for three sequential days prior to oral administration of lovastatin at a dose of 2 mg/kg. Heparinized blood samples (approximately 0.2 mL) were collected alternately in each edge of eye via the oculichorioideae vein under anesthesia by ether at 0.15, 0.5, 1.0, 2.0, 4.0, 6.0, 8.0, 10, 12 and 24 h. Plasma samples were obtained by centrifuge under 4000× *g* to obtain the decanted supernatant, which was stored at −40 °C before analysis.

### 4.7. Evaluation of the Metabolic Activity of Lovastatin in BER-Induced HepG2 Cells

According to our previously study, BER significantly increased the metabolic activity of the CYP 3A4 enzyme in a dose-dependent manner by increasing of mRNA and protein expression [30]. To investigate the induction effect of BER on HepG2 cells, 200 μL of 1 × 10^6^/mL HepG2 cell suspension was placed in a 6-well plate and incubated with 2 mL of culturemedium for 4 days (culture medium was replaced every 2 days). When the cell fusion was approximately 90%, the medium was discarded, and 2 mL of BER (1.0 μg·mL^−1^ prepared in culture medium) was added into three wells, and 2 mL of drug-free culture medium was added into the other three wells. After incubation for three days, the medium was discarded, 2 mL of lovastatin (1.0 μg·mL^−1^ prepared in culture medium) was added into each well prior to incubation for 90 min, then 100 μL of the sample were collected at 0, 30, 60 and 90 min, the sample was added with 50 μL ofice-cold sodium acetate buffer (100 mM; pH 4.5 at room temperature) prior to addition of 150 μL cool acetonitrile (containing 100 ng·mL^−1^ warfarin as internal standard, 4 °C), then vortexed for 2 min and centrifuged at 13,500× *g* for 15 min, the supernatant was placed into a 96-well polypropylene plate for analysis, where the sodium acetate buffer was used to protect the transformation of lovastatin and lovastatin acid [39]. The linearity for the analysis of lovastatin was determined with 100 μL of blank samples (200 μL of HepG2 cell suspension added with 2 mL culture medium) added with 50 μL of ice-cold sodium acetate buffer (100 mM; pH 4.5 at room temperature) and 150 μL cool acetonitrile (containing 100 ng·mL^−1^ warfarin as internal standard, 4 °C) prepared at final concentrations levels of 10, 25, 50, 100, 250 and 500 ng·mL^−1^, QC samples were prepared at final concentrations of 30, 75, 150, 300 ng·mL^−1^ as the sample preparation. The UPLC-MS/MS analysis was performed as well as determination of lovastatin acid. MS/MS condition transitions (*m*/*z*) for lovastatin were 405.35→285.22, the parameters of cone voltage and collision energy were 16 and 12 V, respectively.

### 4.8. Data Analysis

The main pharmacokinetic parameters were calculated by the compartment model using the quantitative pharmacology software DASStatistics Version 1.0 (Mathematical Pharmacology Professional Committee of China, Shanghai, China), the mean plasma concentration–time profile was plotted in the figure using a horizontal axis of time (hours) and a vertical axis of plasma concentration (ng/mL, *n* = 6). All data presentation was in the format of mean and standard deviation (mean ± SD). An independent T test was used for inter-group (lovastatin group and berberine induced group) comparison (SPSS 17.5, IBM SPSSS tatistics software, NewYork, NY, USA). For analysis, aprobability value *p* < 0.05 or *p* < 0.01 were considered to be significant or very significant in the statistics.

## 5. Conclusions

In conclusion, berberine could be an inducer for the metabolism of lovastatin according to the results of our *in vivo* pharmacokinetics study and *in vitro* HepG2 cell metabolism study. According to our previous research on the berberine induction effects on HepG2 cells, it may be relevant to berberine induction of the CYP 450 3A4 enzyme. Meanwhile, these experiments provide an example of a drug metabolism study *in vitro* in relation to a drug pharmacokinetics study *in vivo*.

## Figures and Tables

**Table 1:** The optimized UPLC-MS/MS condition in the MRM model for lovastatin acid and warfarin.

Compound	Molecular Weight	Precursor Ion	Product Ion (*m*/*z*)	Cone (V)	Collision (V)	Retention Time (min)
Lovastatina cid	444.5	445.35	343.26	36	18	2.3
Warfarin	330.10	331.30	273.03	44	22	2.1

**Table 2:** UPLC-MS/MS method validation data for the determination of lovastatin acid in rat plasma (*n* = 6).

Content		Concentration (ng·mL^−1^)			
		Nominal	1	10	100
Recovery (%)		Accuracy	89.2	92.5	94.6
		RSD	6.8	5.7	4.3
Matrixeffect (%)		Accuracy	95.8	96.4	102.7
		RSD	6.4	4.1	6.9
Precision (%)	Inter-day	Accuracy	92.5	94.6	101.5
		RSD	9.6	6.1	4.8
	Inner-day	Accuracy	89.6	98.3	104.2
		RSD	6.5	7.2	4.4
Stability (%)	1cycle	Accuracy	92.2	96.8	102.3
		RSD	8.2	4.6	3.5
	3cycles	Accuracy	90.6	93.1	95.9
		RSD	7.8	6.3	5.4
	12 h post-procedure	Accuracy	95.3	97.9	103.4
		RSD	5.9	4.8	3.3
	12 h under sampler	Accuracy	94.6	97.5	101.6
		RSD	6.8	4.2	4.1

**Table 3:** The main pharmacokinetics parameter between berberine induced prior to dosing with lovastatin and a single dose of lovastatin.

Parameter	Berberine Induced Prior to Lovastatin		Lovastatin	
	Mean	SD	Mean	SD
t_1/2α_ (h)	3.75 *	2.19	2.20	0.41
t_1/2β_ (h)	16.60 **	5.46	2.93	1.32
V (L)	0.24	0.09	0.09	0.05
CL/F (L/h)	0.05	0.02	0.03	0.01
AUC_0–12_ (ng/mL·h)	37.28 **	14.85	91.97	55.01
AUC_0-∞_ (ng/mL·h)	41.17 **	15.98	93.66	55.49
Lagtime (h)	0.03	0.05	0.02	0.05
MRT_0–12_ (h)	7.42	1.41	4.37	0.97
*t*_max_ (h)	1.03	0.59	0.92	0.20
C_max_ (ng/mL)	11.13 **	4.32	47.40	14.39

where *: is the in representation of *p* < 0.05, **: is the in representation of *p* < 0.01. The comparisons between two groups are berberine-induced lovastatin group and lovastatin group.

## References

[1] Xu Y., Wang L., He J., Bi Y., Wang T., Wang L., Jiang Y., Dai M., Lu J., Xu M. (2013). Prevalence and control of diabetes in Chinese adults. JAMA.

[2] Lee C.L., Wang J.J., Kuo S.L., Pan T.M. (2006). Monascus fermentation of dioscorea for increasing the production of cholesterol-lowering agent-monacolin K and anti-inflammation agent-monascin. Appl. Microbiol. Biotechnol..

[3] Park H.J., Kong D., Iruela-Arispe L., Begley U., Tang D., Galper J.B. (2002). 3-Hydroxy-3-methylglutaryl coenzyme A reductase inhibitors interfere with angiogenesis by inhibiting the geranylgeranylation of Rho A. Circ Res..

[4] Jacobsen W., Kirchner G., Hallensleben K., Mancinelli L., Deters M., Hackbarth I., Benet L.Z., Sewing K.F., Christians U. (1999). Comparison of cytochrome P450-dependent metabolism and drug interactions of the 3-hydroxy-3-methylglutaryl-CoA reductase inhibitors lovastatin and pravastatin in the liver. Drug Metab. Dispos..

[5] Mangravite L.M., Engelhardt B.E., Medina M.W., Smith J.D., Brown C.D., Chasman D.I., Mecham B.H., Howie B., Shim H., Naidoo D. (2013). A statin-dependent QTL for GATM expression is associated with statin-induced myopathy. Nature.

[6] Lodge J.W., Fletcher B.L., Brown S.S., Parham A.J., Fernando R.A., Collins B.J. (2008). Determination of lovastatin hydroxy acid in female B6C3F1 mouse serum. J. Anal. Toxicol..

[7] Li M., Fan L.Y., Zhang W., Cao C.X. (2007). Stacking and quantitative analysis of lovastatin in urine samples by the transient moving chemical reaction boundary method in capillary electrophoresis. Anal. Bioanal. Chem..

[8] Liu Y., Zeng B.H., Shang H.T., Cen Y.Y., Wei H. (2008). Bama miniature pigs (*Sus*
*scrofa*
*domestica*) as a model for drug evaluation for humans: Comparison of *in vitro* metabolism and *in vivo* pharmacokinetics of lovastatin. Comp. Med..

[9] Li Z., Seeram N.P., Lee R., Thames G., Minutti C., Wang H.J., Heber D. (2005). Plasma clearance of lovastatin *versus* Chinese red yeast rice in healthy volunteers. J. Altern. Complement. Med..

[10] Donovan J.M., Kisicki J.C., Stiles M.R., Tracewell W.G., Burke S.K. (2002). Effect of colesevelamon lovastatin pharmacokinetics. Ann. Pharmacother..

[11] Prueksaritanont T., Zhao J.J., Ma B., Roadcap B.A., Tang C., Qiu Y., Liu L., Lin J.H., Pearson P.G., Baillie T.A. (2002). Mechanistic studies on metabolic interactions between gemfibrozil and statins. J. Pharmacol. Exp. Ther..

[12] Kivist K.T., Kantola T., Neuvonen P.J. (1998). Different effects of itraconazole on the pharmacokinetics of fluvastatin and lovastatin. Br. J. Clin. Pharmacol..

[13] Lilja J.J., Neuvonen M., Neuvonen P.J. (2004). Effects of regular consumption of grape fruit juice on the pharmacokinetics of simvastatin. Br. J. Clin. Pharmacol..

[14] Yoo D.H., Kim I.S., Van Le T.K., Jung I.H., Yoo H.H., Kim D.H. (2014). Gutmicrobiota-mediated drug interactions between lovastatin and antibiotics. Drug Metab. Dispos..

[15] Keskitalo J.E., Kurkinen K.J., Neuvonen M., Backman J.T., Neuvonen P.J., Niemi M. (2009). No significant effect of ABCB1 haplotypes on the pharmacokinetics of fluvastatin, pravastatin, lovastatin, and rosuvastatin. Br. J. Clin. Pharmacol..

[16] Chen G., Lu F., Xu L.J., Dong H., Yi P., Wang F., Huang Z., Zou X. (2013). The anti-diabetic effects and pharmacokinetic profiles of berberine in mice treated with Jiao-Tai-Wan and its compatibility. Phytomedicine.

[17] Shi R., Zhou H., Liu Z.M., Ma Y., Wang T., Liu Y., Wang C. (2009). Influence of Coptis Chinensis on pharmacokinetics of flavonoids after oral administration of radix Scutellariae in rats. Biopharm. Drug Dispos..

[18] Chen J.L., Zhang Y.L., Dong Y., Gong J.Y., Cui H.M. (2013). CYP 450 enzyme inhibition of berberine in pooled human liver microsomes by cocktail probe drugs. China J. Chin. Mater. Med..

[19] Gurley B.J., Swain A., Barone G.W., Williams D.K., Breen P., Yates C.R., Stuart L.B., Hubbard M.A., Tong Y., Cheboyina S. (2007). *E*ffect of goldenseal (*Hydrastiscanadensis*) and kavakava (*Pipermethysticum*) supplementation on digoxin pharmacokinetics inhumans. Drug Metab. Dispos..

[20] Kong W.J., Wei J., Zuo Z.Y., Wang Y.M., Song D.Q., You X.F., Zhao L.X., Pan H.N., Jiang J.D. (2008). Combination of simvastatin with berberine improves the lipid-lowering efficacy. Metabolism.

[21] Tong N., Zhang J., Chen Y., Li Z., Luo Y., Zuo H., Zhao X. (2012). Berberine sensitizes multiple human cancer cells to the anticancer effects of doxorubicin *in vitro*. Oncol. Lett..

[22] Musumeci R., Speciale A., Costanzo R., Annino A., Ragusa S., Rapisarda A., Pappalardo M.S., Iauk L. (2003). *Berberisaetnensis* C. Presl. extracts: Antimicrobial properties and interaction with ciprofloxacin. Int. J. Antimicrob. Agents.

[23] Quan H., Cao Y.Y., Xu Z., Zhao J.X., Gao P.H., Qin X.F., Jiang Y.Y. (2006). Potent *In vitro* synergism of fluconazole and berberine chloride against clinicalisolates of *Candidaalbican*s resistant to fluconazole. Antimicrob. Agents Chemother..

[24] Tsai P.L., Tsai T.H. (2005). Measurement of unbound pravastatin in rat blood and bile on the perspective of hepatobiliary excretion and its interaction with cyclosporin A and berberine. Anal. Chim. Acta.

[25] Wang Z., Hu H., Chen F., Lan K., Wang A. (2013). Reduced system exposures of total rhein and baicalin after combinatory oral administration of rhein, baicalin and berberine to beagle dogs and rats. J. Ethnopharmacol..

[26] Wu X.C., Li Q., Xin H., Yu A., Zhong M. (2005). Effects of berberine on the blood concentration of cyclosporine A in renal transplanted recipients: Clinical and pharmacokinetic study. Eur. J. Clin. Pharmacol..

[27] Xin H.W., Wu X.C., Li Q., Yu A.R., Zhong M.Y., Liu Y.Y. (2006). The effects of berberine on the pharmacokinetics of ciclosporin A in healthy volunteers. Methods Find Exp. Clin. Pharmacol..

[28] Zhou Y.M., He P., Liu A.M., Zhang L., Liu Y., Dai R. (2011). Drug-drug interactions between ketoconazole and berberine in rats: Pharmacokinetic effects benefit pharmacodynamic synergism. Phytother. Res..

[29] Chi L., Peng L., Hu X., Pan N., Zhang Y. (2014). Berberine combined with atorvastatin downregulates LOX1 expression through the ET1 receptor in monocyte macrophages. Int. J. Mol. Med..

[30] Cui H.M., Zhang Q.Y., Wang J.L., Chen J.L., Zhang Y.L., Tong X.L. (2014). *In*
*vitro* studies of berberine metabolism and its effect of enzyme induction on HepG2 cells. J. Ethnopharmacol..

[31] Chen C., Lin J., Smolarek T., Tremaine L. (2007). *P*-glycoprotein has differential effects on the disposition of statin acid and lactone form sinMDR1A/B knockout and wild-typemice. Drug Metab. Dispos..

[32] Berta E., Harangi M., Zsíros N., Nagy E.V., Paragh G., Bodor M. (2014). Effect of thyroidhormone status and concomitant medication on statin induced adverse effects in hyperlipidemic patients. Pharmazie.

[33] Li J., Volpe D.A., Wang Y., Zhang W., Bode C., Owen A., Hidalgo I.J. (2011). Use of transporter knockdown Caco-2 cells to investigate the *in vitro* efflux of statin drugs. Drug Metab. Dispos..

[34] Wang J., Luzum J.A., Phelps M.A. (2015). Liquid chromatography-tandem mass spectrometry assay for the simultaneous quantification of simvastatin, lovastatin, atorvastatin, and their major metabolites in human plasma. J. Chromatogr. B.

[35] Guidance for Industry: Bioanalytical Method Validation. http://www.fda.gov/downloads/Drugs/Guidances/ucm070107.pdf.

[36] Drug Bank Database DB0022. http://www.drugbank.ca/drugs/DB00227.

[37] FDA guidance Drug Development and Drug Interactions: Table of Substrates, Inhibitors and Inducers. http://www.fda.gov/drugs/developmentapprovalprocess/developmentresources/druginteractionslabeling/ucm080499.htm.

[38] Liu M., Su X., Li G., Zhao G., Zhao L. (2015). Validated UPLC-MS/MS method for simultaneous determination of simvastatin, simvastatin hydroxyl acid and berberine in rat plasma: Application to the drug-drug pharmacokinetic interaction study of simvastatin combined with berberine after oral administration in rats. J. Chromatogr. B Anal. Technol. Biomed. Life Sci..

[39] Ju W.J., Peng K.W., Yang S.Y., Huijing S., Mario S., Zhuo W.M. (2015). A simple protein precipitation-based simultaneous quantification of lovastatin and its active metabolite lovastatin acid in human plasma by ultra-performance liquid chromatography-tandem mass spectrometry using polarity switching. J. Chromatogr. Sep. Technol..

